# Evaluation of the Ion Channel Assembly in a Eukaryotic Cell-Free System Focusing on Two-Pore Domain Potassium Channels K_2P_

**DOI:** 10.3390/ijms24076299

**Published:** 2023-03-27

**Authors:** Jessica Ullrich, Carsten Ohlhoff, Srujan Kumar Dondapati, Anne Zemella, Stefan Kubick

**Affiliations:** 1 Fraunhofer Institute for Cell Therapy and Immunology (IZI), Branch Bioanalytics and Bioprocesses (IZI-BB), Am Mühlenberg 13, 14476 Potsdam, Germany; 2 Institute of Biotechnology, Technische Universität Berlin, Straße des 17. Juni 135, 10623 Berlin, Germany; 3 Institute of Chemistry and Biochemistry, Freie Universität Berlin, Thielallee 63, 14195 Berlin, Germany; 4 Faculty of Health Sciences, Joint Faculty of the Brandenburg University of Technology Cottbus-Senftenberg, the Brandenburg Medical School Theodor Fontane and the University of Potsdam, 14476 Potsdam, Germany

**Keywords:** eukaryotic cell-free protein synthesis (CFPS), cell-free synthesis (CFS), membrane protein synthesis, ion channel, TREK-2, TWIK-1, K_2P_, heterodimerization, oligomerization, protein assembly

## Abstract

Oligomeric ion channels are abundant in nature. However, the recombinant expression in cell culture-based systems remains tedious and challenging due to negative side effects, limiting the understanding of their role in health and disease. Accordingly, in this work, we demonstrate the cell-free synthesis (CFS) as an alternative platform to study the assembly of two-pore domain potassium channels (K_2P_) within endogenous endoplasmic reticulum-derived microsomes. Exploiting the open nature of CFS, we investigate the cotranslational translocation of TREK-2 into the microsomes and suggest a cotranslational assembly with typical single-channel behavior in planar lipid-bilayer electrophysiology. The heteromeric assembly of K_2P_ channels is a contentious matter, accordingly we prove the successful assembly of TREK-2 with TWIK-1 using a biomolecular fluorescence complementation assay, Western blot analysis and autoradiography. The results demonstrate that TREK-2 homodimer assembly is the initial step, followed by heterodimer formation with the nascent TWIK-1, providing evidence of the intergroup heterodimerization of TREK-2 and TWIK-1 in eukaryotic CFS. Since K_2P_ channels are involved in various pathophysiological conditions, including pain and nociception, CFS paves the way for in-depth functional studies and related pharmacological interventions. This study highlights the versatility of the eukaryotic CFS platform for investigating ion channel assembly in a native-like environment.

## 1. Introduction

Potassium channels are the largest and most diverse class of ion channels, with important physiological and pathophysiological roles in the human body. Among these, two-pore domain K^+^ channels (K_2P_) are a family essential for background conductance and the resting membrane potential [1]. These channels are located throughout the body and can be subdivided into six subgroups based on their structure and function. Structurally, they share a homodimerizing conformation, with four transmembrane segments, two pore-forming domains, and a pseudo-tetrameric architecture [2,3]. Recent research has suggested that the oligomerization of the K_2P_ channels precedes ribosomal release in protein biosynthesis due to domain swapping [4,5]. While the homodimeric structure of all K_2P_ channels has been identified, the function and mode of assembly of some channels remain to be elucidated. Intragroup heteromerization within the subgroup has been widely accepted [6,7,8,9]. However, the heterodimerization of different K_2P_ channel subgroups (intergroup) remains controversial, with only a limited number of oligomers and their functionality identified [10,11,12,13].

This study focuses on the TWIK-related potassium channel 2 (TREK-2), a potassium channel involved in pain perception [14]. Only a limited number of inter-, and intragroup heterodimers have been identified for this protein [10,11]. This is associated with the challenges of the heterologous overexpression of ion channels, including cytotoxicity, denaturing purification and solubilization procedures, that disrupt protein assemblies, along with the difficulty in controlling the dynamics of protein complexes [15,16]. These limitations not only restrict research but lead to inconsistent results. Consequently, the understanding of K_2P_ channels with their diverse functions is limited, which in turn restricts the understanding of pathophysiological conditions and their pharmacological interventions [17,18].

Cell-free synthesis (CFS) has attracted attention as a simple and controllable alternative to address the challenges of the functional heterologous overexpression of ion channels. It allows the direct manipulation of protein expression and facilitates the direct characterization and detailed assembly studies by autoradiography and planar lipid bilayer measurements. By using a lysate based on eukaryotic insect cells (*Spodoptera frugiperda* 21, *Sf*21), endogenous endoplasmic reticulum-derived structures (microsomes) are retained, enabling native-like protein maturation [19,20,21]. The modular addition of protein-coding plasmids to the CFS allows a straightforward and defined study of protein assembly. Exploiting the open character of CFS in a system representative of cellular biosynthesis opens new possibilities for the analysis of oligomeric membrane proteins.

Accordingly, we demonstrate the CFS of the functional potassium channel, TREK-2. We validate the homomeric protein assembly in endogenous microsomes in a time-dependent manner, as well as its single-channel activity by planar lipid bilayer electrophysiology. Furthermore, we identify the cotranslational translocation of TREK-2 into microsomes and suggest cotranslational homomer formation. We demonstrate the interfamily heterodimer assembly of TREK-2 and TWIK-1 in a time-dependent manner by a biomolecular fluorescence complementation assay, autoradiography and Western blot analysis. This study qualifies CFS as a platform technology for studying oligomeric protein assembly in a native-like environment and paves the way for its application in pharmacological studies.

## 2. Results

### 2.1. Cell-Free Synthesis of TREK-2 in a Eukaryotic Vesicle-Based System

**Figure 1:**
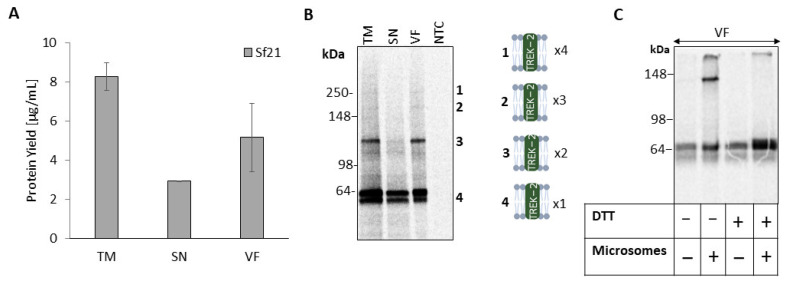
Cell-free synthesis of TREK-2 in *Sf*21-lysate. TREK-2 was synthesized in *Sf*21 lysate and ^14^C labeled. The translation mixture (TM) was fractionated after synthesis by centrifugation in the supernatant (SN) and the vesicular fraction (VF). (**A**) Quantitative analysis was performed by liquid scintillation counting of synthesized TREK-2. Standard deviations were calculated from triplicates. (**B**) Autoradiogram of fractions depicting protein bands of TREK-2 by a non-denatured SDS–PAGE (4–12% Tris-Glycine). A no-template control (NTC) was used as a negative control. (**C**) Autoradiogram of VF synthesized in microsome-containing or -depleted lysate. After synthesis, samples were either reduced (supplemented with dithiothreitol (DTT)) or non-reduced applied to SDS–PAGE (10% Tris–Glycine).

The heterologous overexpression of many membrane proteins, including ion channels, is crucial. Accordingly, in this work, we validate the cell-free synthesis (CFS) of the full-length, homo-oligomeric potassium channel TREK-2. Therefore, a *Sf*21-based CFS (*Spodoptera frugiperda* 21) was performed for 180 min and supplemented with ^14^C-leucine for quantification, yielding a protein concentration of 8,3 µg/mL in the crude translation mixture (TM) (Figure 1a). After synthesis, TM was subdivided by centrifugation into the vesicular fraction (VF) and the supernatant (SN), resulting in a high concentration of TREK-2 in the VF (5.17 µg/mL). Qualitative analysis by non-denatured SDS-PAGE with subsequent autoradiography revealed a monomeric double band at approximately 62,6 kDa in all fractions (Figure 1b). In addition, strong high-molecular-weight bands at 125 kDa were detectable in both TM and VF fractions. The negative control (NTC) did not show any unspecific signal. However, in the TM and VF, additional high-molecular-weight bands were visible at ~180 kDa and ~250 kDa.

TREK-2 channels have previously been found to assemble as dimers linked by disulfide bridges to form an active ion channel [2]. Mimicking the endoplasmic-reticulum (ER) environment is often a crucial prerequisite for membrane protein maturation including folding, assembly and, thus, functionality. As the microsomes are mainly present in the VF after fractionation, we found the 125 kDa band to correspond to disulfide-bridged subunits. Therefore, we removed the microsomes from *Sf*21-lysate by centrifugation prior to CFS to hinder translocation and prevent ER-based maturation of the protein including disulfide bridging (Figure 1c). After synthesis, without microsomes only a monomeric double band was detectable, while dimeric bands were only detectable in the presence of microsomes. When reducing conditions were applied after synthesis, the 125 kDa band disappeared, indicating disulfide bridging and successful dimeric assembly of TREK-2.

The absence of oligomerization in the SN without microsomes indicated that the protein assembly requires the microsomal surrounding for protein maturation.

We were able to confirm the successful synthesis of TREK-2 assembled by disulfide bridges, but were still wondering about the monomeric double band. Previous studies have shown that the insect-based CFS platform facilitates core glycosylations of proteins [22]. We assumed the three described glycosylation sites of TREK-2 as the reason for the double band [2]. To verify this, a deglycosylation assay based on an endoglycosidase that cleaves N-linked glycans between protein core asparagine and the N-acetylamine was performed. As a result, a slight reduction in the broadness of the monomeric band (Figure S1) was revealed. These results indicate that TREK-2 is glycosylated; however, the second monomeric prominent band did not disappear and therefore could not be attributed to the presence of N-glycosylations in the *Sf*21 lysate.

### 2.2. Homomeric TREK-2 Assembly in Endoplasmic-Reticulum-Derived Microsomes

**Figure 2:**
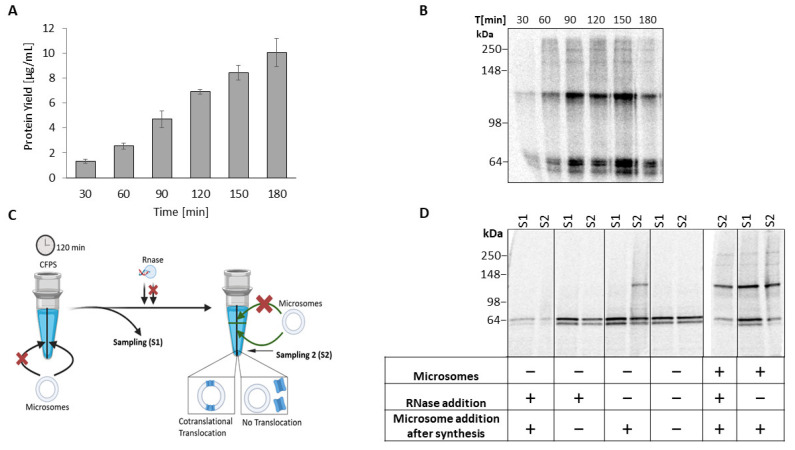
TREK–2 assembly in eukaryotic cell-free synthesis. TREK–2 was synthesized in *Sf*21 lysate and ^14^C labeled. The vesicular fraction (VF) was analyzed. (**A**) Quantitative analysis of time series by liquid scintillation counting. Standard deviations were calculated from triplicates. (**B**) Autoradiogram of time series depicting protein bands of TREK-2 by a non-denatured SDS–PAGE (4–12% Tris–Glycine). (**C**) Schematic diagram of assembly experiment. (**D**) Autoradiogram of assembly experiment depicting protein bands of TREK-2 by a non-denatured SDS-PAGE (4–12% Tris-Glycine) with varied synthesis conditions: with or without microsomes; supplemented microsomes from the beginning or after 120 min; RNase addition. Rearranged: original autoradiogram, Figure S2.

Assessment of protein complexes is challenging, although oligomerization is of utmost importance for functional diversity. Accordingly, we examined the formation of the oligomeric bands in a time-dependent manner. Thereby, a constant increase in the protein yield over time was observed (Figure 2a). Correspondingly, in the autoradiogram, the band intensity increased over time, whereby the dimeric band became already visible after 30 min. After 90 min, additional bands again became apparent at ~187 kDa and ~250 kDa (cf. Figure 1c). This finding suggests early protein assembly during biosynthesis, potentially cotranslational.

To characterize the assembly of TREK-2 during CFS, microsomes were depleted from the *Sf*21 lysate prior to synthesis and the assembly pattern was qualitatively analyzed by autoradiography (Figure 2c,d). After 120 min of synthesis (sampling point S1), only monomeric bands were detectable. In contrast, when using microsome-enriched lysate, oligomeric bands appeared. This finding proves that microsomes, with their native-like ER-based environment, are crucial for the assembly of TREK-2 oligomers. To validate whether unassisted protein integration into the microsomes occurred, CFS was interrupted by RNase addition and subsequently enriched with microsomes (sampling point S2). Consequently, TREK-2 showed no further oligomeric assembly indicating no passive integration. Instead, if CFS proceeded and microsomes were supplemented post-synthesis, a shift from a monomeric band at S1 to a dimeric band at S2 appeared in the autoradiogram. As the integration of membrane proteins into microsomes can occur either co-translationally during protein synthesis or post-translationally, these findings indicate cotranslational translocation and furthermore suggest a cotranslational assembly of the dimeric TREK-2.

Remarkably, the presence of microsomes from the beginning of the synthesis resulted in high-molecular-weight bands, in addition to the monomeric and dimeric bands. However, if microsomes were added post-synthesis, only a dimeric band was detectable. These results correspond to the previous time series (Figure 2b) and additionally suggest the requirement of further protein processing time for higher order oligomer assembly. It should be noted that the absence of microsomes reduced the protein yield, consistent with other studies [23] and thus causes overall weaker protein bands in the autoradiogram.

### 2.3. Analysis of TREK-2 Functionality

**Figure 3:**
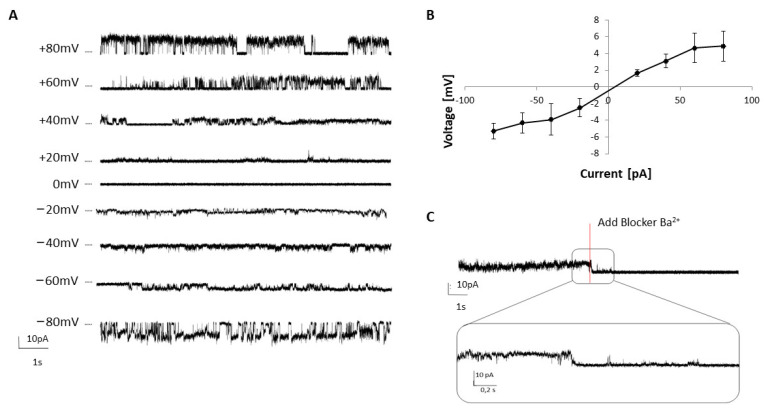
Electrophysiological characterization of cell-free synthesized TREK-2. TREK-2 was synthesized in *Sf*21 lysate and the vesicular fraction was tested by planar lipid bilayer-based electrophysiology (DPhPC lipids and 150 mM KCl in 20 mM Hepes buffer). (**A**) Single-channel recordings of TREK-2 at different voltages (−80 mV to −80 mV). (**B**) Voltage–current ratio at the applied conditions. (**C**) Blocking of a functional TREK-2 channel with 5 mM Ba^2+^.

We proceeded to validate the CFS platform for downstream biochemical and pharmacological applications by assessing the functionality of the synthesized dimeric TREK- 2. Therefore, we tested the VF using planar lipid bilayer-based electrophysiology. The synthesized protein was reconstituted into a previously coated DPhPC membrane, and the changed gating behavior at varied voltage potentials was analyzed. To avoid potential electrogenic activity of endogenous protein that may be present in the microsomes, a negative control without plasmid (NTC) was measured first, showing no activity. However, the synthesized VF containing TREK-2 exhibited single-channel behavior, with a typical opening (activation) and closing (inactivation) at applied voltage ranging from −80 mV to +80 mV (Figure 3a). Representative amplitude histograms from channel openings are depicted in Figure S3. The protein displayed an inward potassium current (Figure 3b), with events clearly assigned to open and closed states of the protein. A single-channel conductance of 88.16 ± 24.42 pS at −40 mV (n > 25) and 69.11 ± 13.29 pS at +40 mV (n > 7) was detectable. An experiment with supplemented barium chloride demonstrated the specificity of the measured activity. The current was continuously recorded and after 2 min, Ba^2+^ (5 mM, 40 mV) was added. With the addition of the blocking agent, the detected single-channel activity decreased drastically to the baseline level, corresponding to the closed state of TREK-2 (Figure 3c). Hence, the single-channel activity can be specifically controlled by barium ions indicating the successful synthesis of functional TREK-2.

### 2.4. Heterodimeric Assembly of TREK-2 and TWIK-1

Detecting protein oligomerization *in vivo* is challenging due to low yields, unstable protein–protein interactions and disrupting purification procedures for downstream applications. However, within the K_2P_ channels, previously homo- and heterooligomers have been found to promote functional diversity of the protein family in the human body [5,8,10,11,24]. Since interfamily heterodimers have given diverse results in the past, we wondered whether the eukaryotic CFS platform also promotes heterodimerization within the K_2P_ channels. Accordingly, we investigated the intergroup heterodimerization of TWIK-1 and TREK-2, which are both in vivo expressed in brain and gastrointestinal tissues.

TWIK-1 is a difficult protein to express in heterologous systems due to low yields [11]. Figure S4 shows that after CFS, a monomeric double band (39 kDa) and a dimeric band (78 kDa) were detectable in the autoradiogram, indicating successful synthesis of this dimerizing K_2P_ channel (Figure S4). The dimeric band was found in TM and VF, supporting that a membrane environment for post-translational protein maturation, such as disulfide bridging and core glycosylation, is required for TWIK-1. The monomeric double band in the VF was further investigated by a deglycosylation assay, which showed the removal of the upper-molecular-weight band. As TWIK-1 has one N-glycosylation site [25], this finding suggests that the protein translocates and matures in the microsomes.

**Figure 4:**
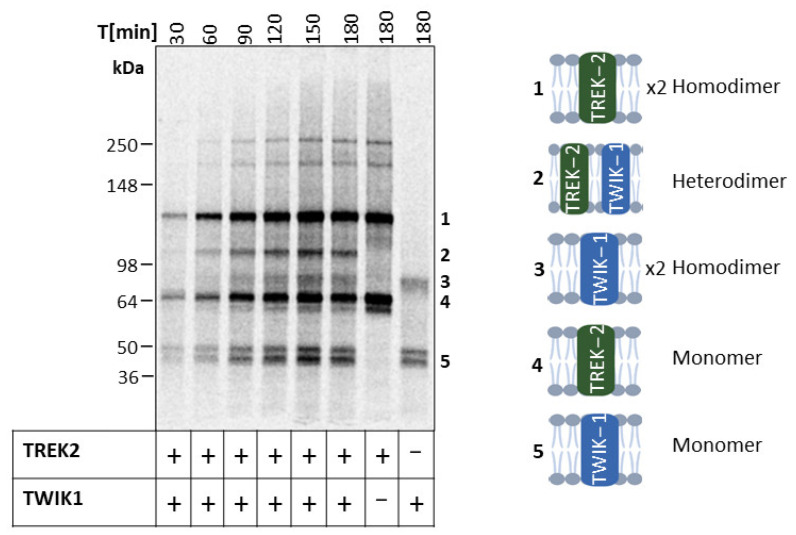
Heterodimerization of TWIK-1 and TREK-2 in cell-free synthesis. Individually and coexpressed subunits of TREK-2 and TWIK-1 in *Sf*21 lysate. Autoradiogram depicting ^14^C-labeled protein bands after synthesis of vesicular fractions (SDS-PAGE 4–12% Tris-Glycine).

We coexpressed TWIK-1 and TREK-2 in a time-dependent manner to investigate potential heterodimeric assembly by autoradiography. The single expression of TREK-2 and TWIK-1 displayed the expected molecular-weight-band pattern. However, after 30 min of coexpression, bands corresponding to TREK-2 and TWIK-1 monomers, as well as TREK-2 dimers were observed. The protein yield increased as protein synthesis progressed (Figure S5), which was also visible in the increasing band intensity in the autoradiogram (Figure 4). Notably, the high-molecular-weight bands at ~180 kDa and 250 kDa reappeared after 60 min in the single expression of TREK-2 and coexpression with TWIK-1 (cf. Figure 1b and Figure 2b,d). The dimeric band of TWIK-1, on the other hand, only became visible after 90 min. However, after 60 min, an additional band appeared at approximately 103 kDa, which was identified as a heterodimer of TREK-2 and TWIK-1 by Western blot analysis (Figure S6).

**Figure 5:**
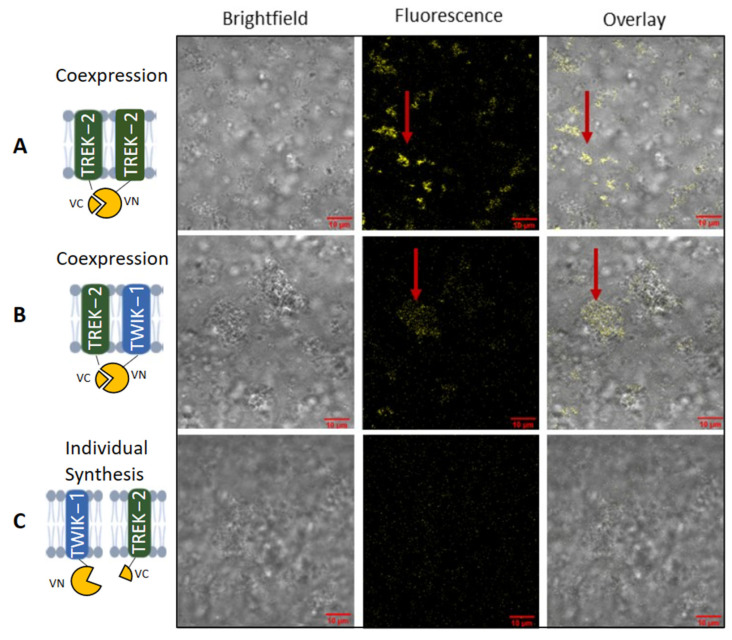
Heterodimerization of TWIK-1 and TREK-2. Coexpression of TREK-2 and TWIK-1 fused with venus split fragments (VN and VC) and expressed in *Sf*21 lysate for a biomolecular fluorescence assay. A detectable yellow fluorescence is produced if split fragments are complemented. Fluorescence signal was detected by confocal microscopy. (**A**) Individual synthesis of TREK-2 venus constructs as positive control. (**B**) Coexpression of TREK-2 and TWIK-1 venus constructs. (**C**) Individual synthesis of TREK-2 and TWIK-1 venus constructs with mixing post-synthesis.

Additionally, we conducted a bimolecular fluorescence complementation assay (BiFC) followed by confocal microscopy (Figure 5). Therefore, the split fragments of venus (VN and VC) were fused to TWIK-1 and TREK-2. If proteins have a distance of less than <7 nm, the fragments can complement and assemble into a yellow fluorescent protein [11]. We detected the expected strong fluorescence for the TREK-2 homodimer (Figure 5a), which was colocalized with the microsomal structures of the brightfield image. This finding supports the essentiality of translocation into the microsomes for protein assembly. The coexpression of TWIK-1 and TREK-2 also resulted in a fluorescence signal (Figure 5b), albeit with lower intensity, consistent with the intensity of the oligomeric band pattern observed in the autoradiogram (Figure 4). In contrast, the individual expression of the constructs did not yield any fluorescence (Figure 5c). To ensure specificity, we synthesized the proteins individually and mixed them post-synthesis to prevent complementation of the venus protein resulting in no fluorescence.

Concludingly, the CFS system facilitates the assembly of heterodimeric K_2P_ channels and indicates the directed, co-post-translational heterodimeric assembly of TREK-2 and TWIK-1 within the ER environment.

## 3. Discussion

Ion channels play a major role in many processes in the human body, making their regulation crucial for pathophysiological conditions and their pharmacological intervention. As the heterologous overexpression of ion channels and their protein complexes is challenging due to cytotoxicity, unstable protein–protein interactions and low protein yields, cell-free synthesis (CFS) has proved a versatile alternative [21,26,27]. In this study, we qualify CFS for the assembly and functional synthesis of the K_2P_ channel TREK-2 in a eukaryotic microsome-containing lysate derived from *Sf*21 cells (Figure 1).

A prerequisite for the functionality of K_2P_ channels is their assembly into functional dimers by disulfide bridging [2,3]. We found protein bands in the autoradiogram corresponding to dimeric TREK-2 (~125 kDa) (Figure 1). Furthermore, we detected the native-like environment of the microsomes to be required for disulfide bridging and assembly, as only the fractions containing microsomal structures (TM and VF) showed oligomerization, while SN revealed only monomeric bands. This finding was further supported by syntheses performed in the absence of microsomal structures, where no dimeric bands were detectable. We furthermore detected the translocation of the protein into the microsomes to be cotranslational (Figure 2) and not to result only from passive integration, in accordance with other studies of cell-free synthesis [28]. We revealed the dimeric assembly to occur early (30 min) during biosynthesis, suggesting that it is cotranslational [4,5].

Unexpectedly, the autoradiogram revealed further weak bands in the microsome-containing vesicular fraction (VF) at 180 kDa and 250 kDa. Based on their molecular weight we suspect these bands to result either of a tetramer or dimer–dimer formation with intermediate trimeric forms of assembly, as reported for other classical potassium channels [29,30]. Another explanation could be protein aggregation as it is a major issue of heterologous protein expression. However, we consider this unlikely as we observed defined bands all over our experiments at the molecular weight corresponding to the potential dimer–dimer and monomer–dimer formations. If the bands were resulting of aggregation, we would also expect to find a similar pattern in the synthesis of TWIK-1, but we cannot determine such bands in the autoradiograms (Figure 5 and Figure S4). Considering that the weak bands only became visible after 90 min of CFS (Figure 2b), we hypothesize that a sufficient amount of protein is necessary for oligomeric assembly, which may pose challenges to detect these interactions in cell culture-based approaches. Furthermore, the detected single-channel activity indicates a correct assembly and conformation of the protein and thus contradicts protein aggregation.

According to our findings, TREK-2 undergoes N-glycosylation, but the observed shift in the apparent molecular weight was less pronounced than in previous reports [2]. We concluded that only minor glycosylation occurred, which did not impact its functionality. We hypothesize that TREK-2 glycosylation is naturally necessary for proper cell trafficking, which may be less crucial in the CFS platform due to the absence of plasma trafficking [31]. Remarkably, we detected a monomeric double band in the autoradiogram which was not due to the N-glycosylations, aswe had observed for other proteins in previous CFS-based studies [22,32] (Figure S1). Earlier studies of TREK-2 have shown a wide variance of single-channel behavior [33,34] associated with an alternative translation start due to the skipping of the first start codon by the ribosomal complex [35], resulting in TREK-2S with a long N-terminus and small conductance level, and TREK-2L with a shorter N-terminus due to downstream ATI and a large conductance level. These ATIs can account for the range of conductance levels spanning from ~52 pS to ~220 pS, previously observed in COS-7, HEK293, HeLa cells and *xenopus oocytes* [33]. Accordingly, the observed monomeric double band in the autoradiogram could be explained by the ATI, with the upper band corresponding to TREK-2S being more intense. This hypothesis is supported by the detected single-channel activity (Figure 3) with a mild inwardly rectifying current–voltage relation andconductance levels of 88.16 ± 24.42 pS at −40 mV and 69.11 ± 13.29 pS at +40 mV.

Evidence is mounting that inter- and intrafamily heterodimers within the K_2P_ channels enable functional diversity of the proteins [5,8,10,11,13,24]. However, for some K_2P_ channels, controversial results have been reported and interfamily heterodimers remain to be elucidated [5]. We investigated if heterodimerization indeed occurs in the eukaryotic CFS. First, we validated the individual synthesis of TWIK-1, which was reported to be especially challenging in cell-culture-based approaches due to low protein yields [11]. Using CFS, we observed monomeric (39 kDa) and dimeric (78 kDa) bands in the autoradiogram (Figure S4), indicating the assembly of TWIK-1 in the TM and VF. This is consistent with previous findings, as assembly requires disulfide bridging and thus the ER surrounding of the microsomes [25].

Previous studies have shown that TWIK-1 can form interfamily heterodimers with TASK [12,36], TRAAK [11] and TREK-1 [11]. However, the heterodimerization of TWIK- 1 and TREK-2 was described only once as a side finding in HEK293T cells [11] and has not been further investigated, although these proteins are both localized in brain tissues. In our coexpression experiments of TWIK-1 and TREK-2, we observed, by autoradiography, the manufacturability of the homodimeric proteins (Figure 4). However, we also detected a band corresponding to the apparent molecular weight of heterodimer formation (103 kDa). This finding was further supported by Western blot analysis (Figure S6) and a biomolecular fluorescence complementation assay (BiFC) (Figure 5). The BiFC assay revealed a strong fluorescence for coexpressed TWIK-1 and TREK-2, indicating the successful complementation of the fused venus split fragments. However, when the proteins were individually synthesized and translocated into different microsomes, no fluorescence was detected upon post-synthesis mixing. The band intensity in the autoradiography (Figure 4) and the fluorescence signal (Figure 5) of the BiFC suggest preferential self-assembly over heterodimeric assembly, which is consistent with other findings [37]. Based on time-dependent CFS, we propose a directed heterodimerization of the fully assembled TREK-2 and the nascent TWIK-1. As heterodimerization was identified to increase functional diversity within the K_2P_ channels, in a next step, an in-depth functional study would be required to determine the biological relevance. A possible high-throughput approach for such studies could be the combination of BiFC, CFS and optical electrophysiology, which would enable the parallel and variable screening of protein–protein interactions and their functionality in a native-like microsome-based environment [38,39].

In conclusion, our findings demonstrate the versatility of CFS as a platform for the functional characterization of K_2P_ channels and, in a broader perspective, membrane protein complexes in a native-like environment. This opens up new opportunities for studying K_2P_ heterodimers and their pathophysiological role in pain and nociception, as well as for other disease-relevant oligomers. Moreover, our results highlight the potential of CFS for in-depth pharmaceutical studies in this area.

## 4. Materials and Methods

### 4.1. Batch-Based Cell-Free Synthesis

The lysate was prepared as previously described [20]. Protein synthesis was performed in a coupled batch mode with a reaction made of 40% (*v*/*v*) translationally active *Sf*21 cell lysate, amino acids (complete 100 µM, Merck, Darmstadt, Germany), Mg(OAc)_2_ (f.c. 3.9 mM, Merck), KOAc (f.c. 150 mM, Merck), spermidine (f.c. 0.25 mM, Roche, Grenzach, Germany), T7 RNA polymerase (1 U/mL, Invitrogen, Waltham, MA, USA), UTP (0.3 mM, Roche), CTP (0.3 mM, Roche) and 0.1 mM of the cap analogue m7G(ppp)G. To provide energy, a regeneration system containing creatine phosphate (20 mM, Roche), creatine phosphate (f.c. 20 mg/mL, Roche), ATP (1.75 mM, Roche) and GTP (f.c. 0.3 mM, Roche) was added to the reaction. Furthermore, polyG primers (f.c. 10 µM, IBA, Göttingen. Germany) and radioactive ^14^C-leucine (f.c. 50 µM, specific radioactivity 66.7 dpm/pmol, Perkin Elmer, Baesweiler, Germany) were used.

The plasmids used were synthesized de novo at biocat (Biocat, Heidelberg, Germany). The human TREK-2 sequence (uniprot: P57789) was fused with an additional internal ribosome entry site (IRES) of the cricket paralysis virus (CrPV). TWIK-1 was designed accordingly (uniprot: O00180).

In the negative control (NTC), the plasmid was replaced by bidestilled water. Synthesis was performed in a thermomixer (Eppendorf, Hamburg, Germany) at 27 °C for the depicted time at 600 rpm. After synthesis, fractionation was performed by centrifuging the samples at 16,000× *g*, for 10 min at 4 °C. The resulting vesicular fraction (VF) was resuspended in PBS and supplemented if marked during the experiment. For syntheses without microsomes, the lysate was centrifuged prior to synthesis at 16,000× *g*, for 15 min at 4 °C, and the supernatant (SN) was used for synthesis. For time series, the samples were stored in liquid nitrogen while the synthesis proceeded. Where depicted, RNase A (5 mg/mL, Qiagen, Venlo, The Netherlands) supplementation was performed.

### 4.2. Quantitative and Qualitative Analyses of Synthesized Proteins

For quantitative analysis, synthesized proteins were analyzed based on the integration of ^14^C-labelled leucine in the cell-free reaction and subsequent hot trichloroacetic acid (TCA; Carl Roth) precipitation and scintillation counting as described previously [32]. The 3 µL samples were precipitated using 10% TCA (*v*/*v*) offset with 2% (*v*/*v*) casein hydrolysate (Carl Roth, Karlsruhe, Germany), boiled for 15 min at 80 °C and cooled down afterwards for 30 min on ice. Using a vacuum filtration system, non-incorporated ^14^C-leucine was removed while the incorporated ^14^C-leucine was determined using liquid scintillation counting (Hidex 600SL, Hidex, Mainz, Germany). The protein concentration was calculated based on the molecular weight, the number of leucines and the specific radioactivity. For coexpressed proteins, the sum of the molecular weight and the number of leucines was used for calculation. The analysis was performed in triplicates and error bars represent the standard deviation.

For qualitative analysis, SDS-PAGE was performed under reducing (supplemented with 50 mM DTT, Applichem, Darmstadt, Germany) and non-reducing conditions with associated autoradiography. Samples were mixed with LDS buffer and loaded on 4–12%, 6%, or 10% Tris-Glycine Gel (Thermo Fisher Scientific, Waltham, MA, USA), and run for 55 min at 160 V, at room temperature, then stained, dried at 70 °C (Unigeldryer 3545D, Unequip Laborgerätebau- und Vetreiebs GmbH, Planegg, Germany) and incubated in a phosphor screen for several days (GE Healthcare, Munich, Germany). The radiolabeled protein was visualized using an Amersham RGB Imager (GE Healthcare). SDS gels were blotted on a PVDF membrane using the iBlot Dry Blotting System (Invitrogen). The membrane was blocked over night with 5% BSA and washed 3 times before the primary antibody was added (#293332, Santa Cruz Biotechnology (Dallas, Texas, USA) (1:1000) in TBS with 1% BSA and 0.1% Tween-20). After 1.5 h, the secondary antibody was added anti-mouse IgG, HRP linked (Cell signaling (Leiden, The Netherlands) (1:3000) in TBS with 1% BSA and 0.1% Tween-20) was added. Membranes were processed using WesternBrightECL HRP substrate (Advansta, San Jose, CA, USA) and an Azure Western C600 Imaging System (Azure Biosystems, Dublin, CA, USA).

### 4.3. The Deglycosylation Assay

A deglycosylation assay was performed using peptide N-glycosidase F, (PNgase F, New Englans Biolabs, Ipswich, MA, USA) on ^14^C-leucine-labelled protein. The assay was performed according to the manufacturer’s instructions using 5 µL of the VF and analyzed in a reducing SDS-PAGE (50 mM DTT) and the according autoradiogram.

### 4.4. Electrophysiological Recordings

Electrophysiology was measured as described previously [21]. The VF sample was applied, and the bilayer formation process was computer controlled by Element Data Reader software (Elements S.R.L., Censena, Italy). Lipid bilayers were formed using DPhPC (Avanti Polar Lipids) dissolved in octane (10 mg/mL, Sigma Aldrich, St. Louis, MO. USA). Experiments were performed in 150 mM KCl (Merck) and 10 mM HEPES (Sigma Aldrich) at pH 7.2. After 20 min of incubation, the measurements were started with various voltage steps (−80 mV to 80 mV), with a sampling rate of 20 kHz. The collected data were analyzed (Clampfit, Molecular Devices, San Jose, CA, USA), and filtered with a low-pass filter at 1 kHz (Bessel filter). Blocking experiments were analyzed accordingly with 5 mM barium chloride applied at +40 mV after 2 min activity.

### 4.5. The Biomolecular Fluorescence Assay

Split venus fragments were fused based on [40]. Samples were either individually or coexpressed. After synthesis, samples could maturate for 2 h at room temperature before microscopy. Split fusion proteins were visualized by confocal laser scanning microscopy (CLSM, LSM 510, Carl Zeiss Microscopy, Jena, Germany) equipped with a Plan-Apochromat 63x/1.4 oil objective and excited with an argon laser at 488 nm. After passing a long-pass filter (LP 505), the emitted light was captured using a photomultiplier.

## Data Availability

The data presented in this study are available on request from the corresponding author.

## Supplementary Materials

The following supporting information can be downloaded at: https://www.mdpi.com/article/10.3390/ijms24076299/s1.
