# Supplementary material for: Evaluation of the Ion Channel Assembly in a Eukaryotic Cell-Free System Focusing on Two-Pore Domain Potassium Channels K2P
Source: Int J Mol Sci. 2023 Mar 27;24(7):6299. doi: 10.3390/ijms24076299 (PMC10094441; doi:10.3390/ijms24076299)
Supplement: Supplementary file 1 [file ijms-24-06299-s001.zip › ijms-2285765-supplementary.pdf]

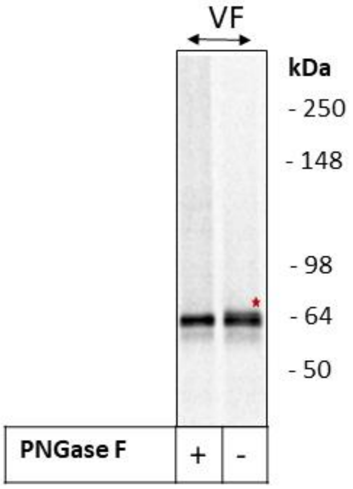

Figure S1. Cell-free synthesis of TREK-2. The product of cell-free synthesis (TM) was fractionated by centrifugation in the supernatant (SN) and the vesicular fraction (VF. Autoradiograph of denatured 4- 12 % SDS-Gel of TREK-2 treated with the endoglycosidase PNGase F. The non-deglycosylated sample is marked with an asterisk.

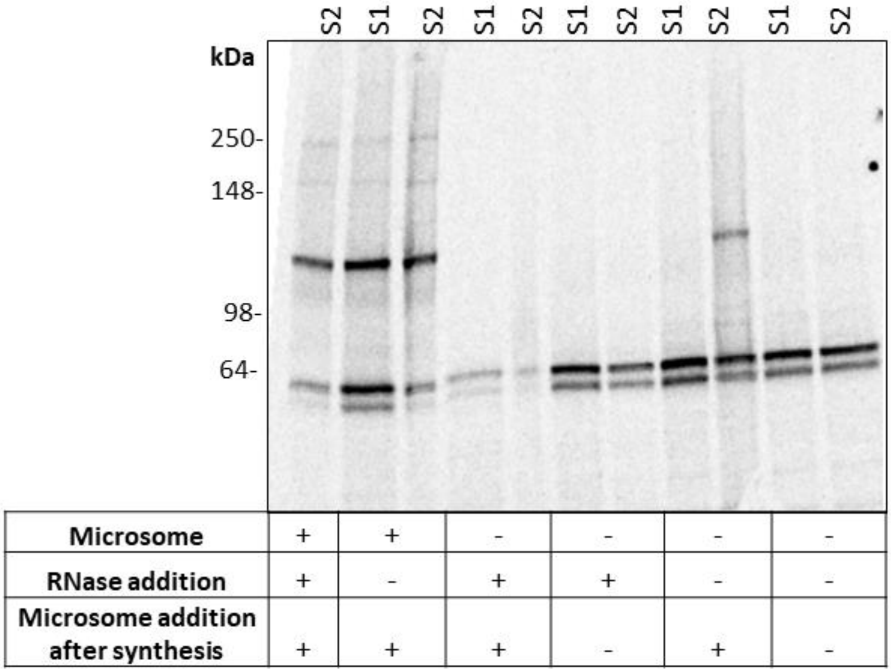

**Microsome**	+	+	-	-	-	-
**RNase addition**	+	-	+	+	-	-
**Microsome addition after synthesis**	+	+	+	-	+	-

Figure S2. CFS was supplemented with 14C-leucine. Synthesis was performed in microsome-containing or -depleted lysate and sampled according to figure 2, c. Autoradiograph of samples is depicted (compare figure 2,d) as applied. For a better understanding the lanes in figure 2, d were rearranged.

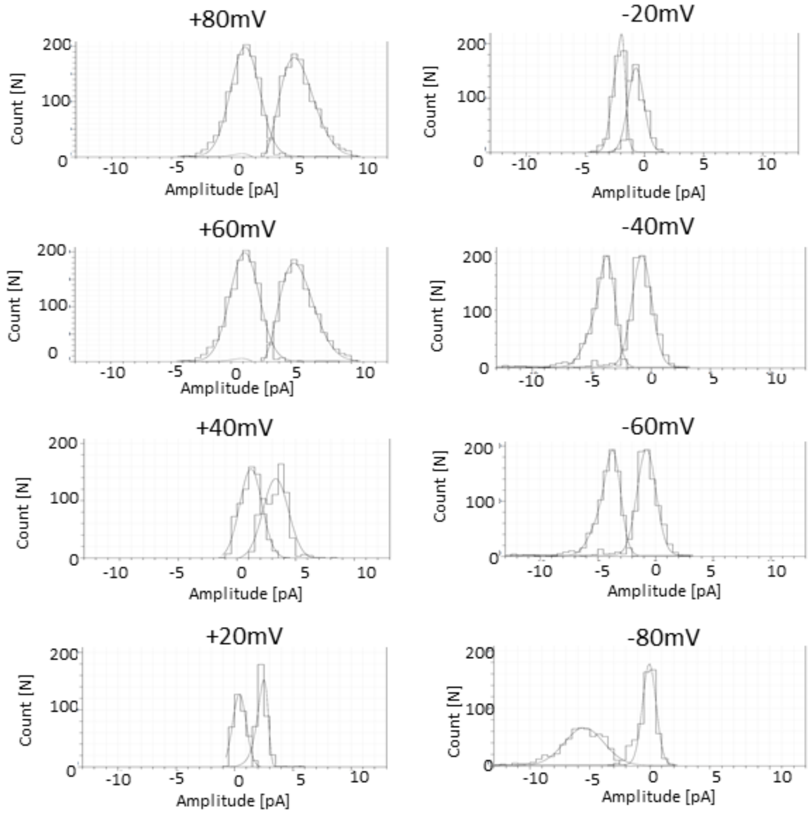

Figure S3. Electrophysiological Characterization: Measurements were performed using planar lipid bilayer electrophysiology, DPhPC lipids and 150 mM KCl in 20 mM Hepes buffer**.** Histograms showing the amplitudes (pA) of the TREK-2 currents against counts of current amplitudes at applied voltages.

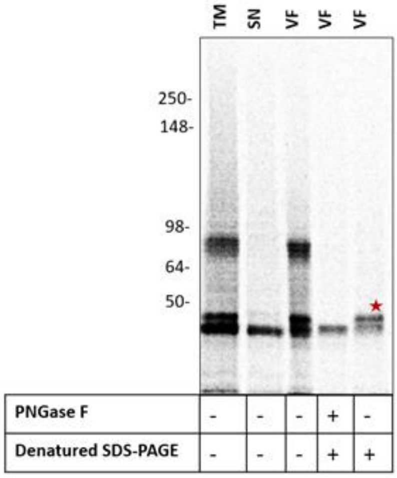

Figure S4. Cell-free synthesis of TWIK-1**.** The product of cell-free synthesis (TM) was fractionated by centrifugation in the supernatant (SN) and the vesicular fraction (VF). Samples were labeled with 14C-leucine. Autoradiograph of 4-12 % Tris-glycine SDS-Gel of TWIK-1 and the corresponding autoradiograph. Samples were deglycosylated (PNGase F) and reduced as indicated. The non-deglycosylated sample is marked with an asterisk.

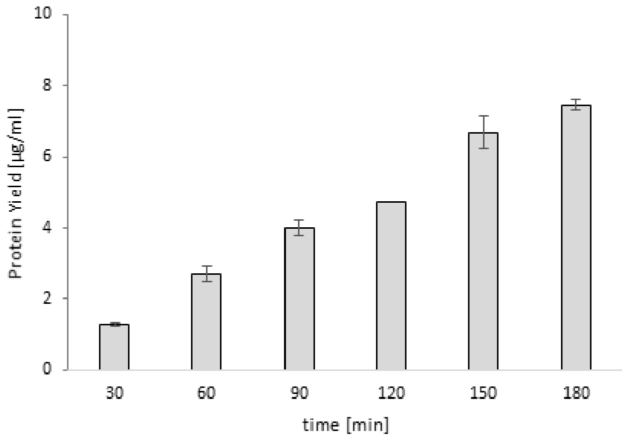

Figure S5. Cell-free coexpression of TWIK-1 and TREK-2: Proteins were coexpressed and labeled with ^{14}C-leucine. Quantitative analysis of TWIK-1 and TREK-2 coexpression over time. Standard deviations were calculated from triplicates.

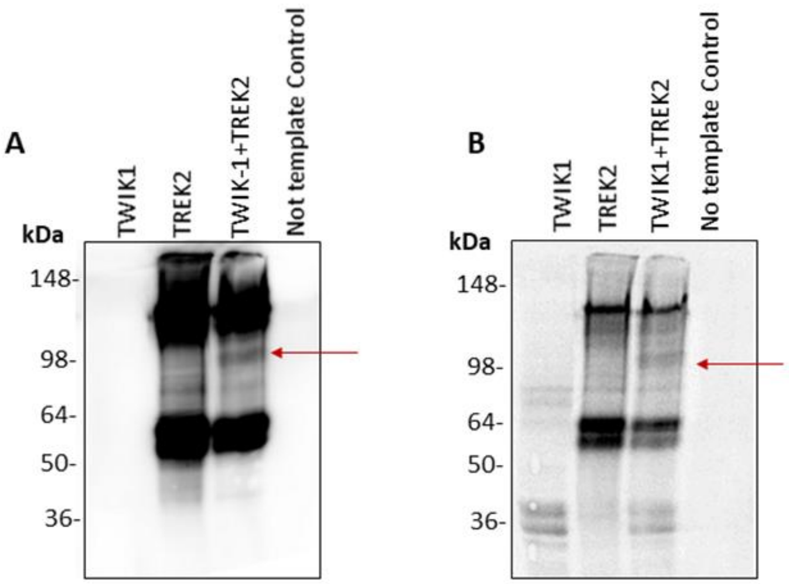

Figure S6. Immunodetection of TREK-2 and TWIK-1 coexpression (A) Western-Blot of coexpression. (B) Corresponding autoradiograph of western-blotting membrane.
